# Dysbiosis of gut microbiota in COVID-19 is associated with intestinal DNA phage dynamics of lysogenic and lytic infection

**DOI:** 10.1128/spectrum.00998-24

**Published:** 2024-12-10

**Authors:** Aya Ishizaka, Azumi Tamura, Michiko Koga, Taketoshi Mizutani, Seiya Yamayoshi, Kiyoko Iwatsuki-Horimoto, Atsuhiro Yasuhara, Shinya Yamamoto, Hiroyuki Nagai, Eisuke Adachi, Yutaka Suzuki, Yoshihiro Kawaoka, Hiroshi Yotsuyanagi

**Affiliations:** 1 Division of Infectious Diseases, Advanced Clinical Research Center, the Institute of Medical Science, the University of Tokyo, Tokyo, Japan; 2 Center for Emergency Preparedness and Response, National Institute of Infectious Diseases, Tokyo, Japan; 3 AIDS Research Center, National Institute of Infectious Diseases, Tokyo, Japan; 4 Division of Virology, Department of Microbiology and Immunology, Institute of Medical Science, the University of Tokyo, Tokyo, Japan; 5 International Research Center for Infectious Diseases, Institute of Medical Science, the University of Tokyo, Tokyo, Japan; 6 The Research Center for Global Viral Diseases, National Center for Global Health and Medicine Research Institute, Tokyo, Japan; 7 The University of Tokyo Pandemic Preparedness, Infection and Advanced Research Center, the University of Tokyo, Tokyo, Japan; 8 Department of Infectious Diseases and Applied Immunology, IMSUT Hospital of Institute of Medical Science, the University of Tokyo, Tokyo, Japan; 9 Department of Computational Biology and Medical Sciences, Graduate School of Frontier Sciences, the University of Tokyo, Chiba, Japan; 10 Influenza Research Institute, Department of Pathobiological Sciences, School of Veterinary Medicine, University of Wisconsin–Madison, Madison, Wisconsin, USA; National Institutes of Health, Bethesda, Maryland, USA

**Keywords:** COVID-19, SARS-CoV-2, gut microbiome, bacteriophages

## Abstract

This study compared intestinal DNA phage dynamics and gut microbiota changes observed at the onset of coronavirus disease 2019 (COVID-19). The study participants included 19 healthy individuals and 19 patients with severe acute respiratory syndrome coronavirus 2 infection. Significant differences were observed in the diversity of the intestinal DNA virome after the onset of COVID-19 compared with that in healthy individuals. Classification by their tail morphology resulted in the order Caudovirales, a double-stranded DNA phage, accounting for >95% of all participants. In classifying phages based on host bacteria, a decreased number of phages infecting mainly the Clostridia class was observed immediately after the onset of COVID-19 and recovered over time. After the onset of COVID-19, two distinct movement patterns of intestinal phages and their host bacteria were observed: phage- and bacteria-predominant. The abundance of obligate anaerobes, such as *Clostridium_sense_strict_1*, *Fusicatenibacter*, and *Romboutsia*, and the phages hosting these bacteria decreased immediately after the onset of COVID-19, and faster phage recovery was observed compared with bacterial recovery. In contrast, the genus *Staphylococcus*, a facultative anaerobic bacterium, increased immediately after the onset of COVID-19, whereas the phages infecting *Staphylococcus* decreased. Furthermore, immediately after the onset of COVID-19, the percentage of lytic phages increased, whereas that of temperate phages decreased. These observations suggest that the gut microbiota dysbiosis observed immediately after the onset of COVID-19 may be linked to phage dynamics that control gut microbiota and may also affect the recovery from dysbiosis.

**IMPORTANCE:**

Bacteriophages infect and replicate with bacteria and archaea and are closely associated with intestinal bacteria. The symbiotic relationship between gut microbiota and bacteriophages is of interest, but it is challenging to study their dynamics in the human body over time. SARS-CoV-2 infection has been reported to alter the gut microbiota, which is involved in gut immune regulation and pathophysiology, although changes in the intestinal phages of patients with SARS-CoV-2 and their dynamic relationship with the gut microbiota remain unclear. SARS-CoV-2 infection, which follows a transient pathological course from disease onset to cure, may provide a reliable model to investigate these interactions in the gut environment. Therefore, this study aimed to elucidate the correlation between gut microbiota and intestinal DNA virome dynamics in COVID-19 pathogenesis. This study found that the dysbiosis observed in SARS-CoV-2 infection involves a growth strategy that depends on the phage or bacterial dominance.

## INTRODUCTION

Severe acute respiratory syndrome coronavirus 2 (SARS-CoV-2) has caused a pandemic of the novel coronavirus infection (coronavirus disease 2019) [COVID-19]), resulting in 773 million infections as of December 2023 (<https://covid19.who.int/>). The main symptoms of COVID-19 include fever, cough, fatigue, and dyspnea; however, several gastrointestinal symptoms, such as diarrhea, nausea, and vomiting, have also been reported (1, 2). Moreover, SARS-CoV-2 RNA has been extensively detected in stools, suggesting that the gastrointestinal tract and lung tissues are sites for viral replication (3).

The disruption of intestinal microbiota has been associated with various chronic diseases such as inflammatory bowel disease, diabetes, and Parkinson’s disease (4). Alterations in the composition of the intestinal microbiota have also been observed in the context of viral infections (5), and intestinal microbiota changes have also been observed in COVID-19 (6–8) and have been shown to be more pronounced in immunocompromised individuals (9). Previous studies have shown that opportunistic pathogens such as *Streptococcus*, *Rothia*, *Veillonella*, and *Actinomyces* were increased in COVID-19 patients (10). In contrast, butyrate-producing bacteria of the Lachnospiraceae and Ruminococcaceae families were decreased (11). Notably, a decrease in beneficial symbiotic bacteria, mainly from the Firmicutes phylum, has been reported, indicating a rapid change in the intestinal environment after disease onset (6–8). Alterations in the gut microbiota persist long after healing (8, 11, 12) and have been reported to correlate with disease severity but have also been implicated in the development of post-acute sequelae of COVID-19, called long-COVID (9, 13).

Intestinal viruses and microbiota are largely found in the digestive system and are crucial in immune regulation and intestinal tract functioning (14). Bacteriophages (phages) are the most abundant intestinal viruses (14). Phages target and replicate within bacteria and archaea and are found in large numbers in several environments (15). Notably, most phages possess a DNA genome and typically infect specific bacterial species or strains (16). However, some phages have a broader host range and can infect multiple bacterial species (16). Phages are also classified based on their life cycle, including the lytic and lysogenic cycles (15). In contrast, during the lysogenic cycle, phages integrate into the bacterial genome and remain dormant as prophages until triggered. Temperate phages have both lytic and lysogenic cycles, whereas lytic phages have only the lytic cycle. Temperate phages use several mechanisms to detect bacterial populations and determine their life cycles, including the SOS response, quorum sensing, and a communication system called “Arbitrium” (17–19). Analysis of the intestinal DNA virome (phageome) has revealed individual differences in the composition of gut phages and that most phages are unique to individuals (20, 21). Research on the intestinal DNA virome in diseases has found that individuals with inflammatory bowel disease have more intestinal phages than healthy individuals (22) and that patients with type 2 diabetes mellitus have altered intestinal phages (23, 24).

The lack of adequate viral databases is a significant challenge in virome analysis (25). Furthermore, much is unknown about the phages that account for a large portion of the gut DNA virome, known as viral dark matter (26). Notably, most double-stranded DNA phages are classified in the order Caudovirales (27); however, they are traditionally classically at the family level (Myoviridae: contracting long tails; Siphoviridae: non-contracting long tails; Podoviridae: non-contracting short tails) based on their morphological classification. The reclassification based on genomic similarity has yet to be established (28). To address this database deficiency, an intestinal DNA virus database (Metagenomic Gut Virus [MGV] catalog) was constructed using publicly available metagenomic data derived from human stool specimens to solve this database deficiency (29). The MGV catalog is based on species identification using the whole-genome sequence similarity. The MGV Catalog contains 189,680 viral genomes and 54,118 species-level viral operational taxonomic units (vOTUs) identified using the average nucleotide identity (ANI) method based on the whole-genome sequence similarity (30). In addition, the MGV catalog assigns a viral taxonomic name by the International Committee on Taxonomy of Viruses (ICTV), virulent or temperate scores that predict phage classification based on their life cycles (31) and predicted phage host information, which improves database performance (29).

Presently, research on intestinal phages and their relationship with alterations in gut microbiota is limited. Since patients with COVID-19 have shown alterations in their gut microbiota, reportedly linked to disease pathogenesis, the SARS -CoV-2 infection, with its transient and significant impact on the gut microbiota, is an effective model for investigating the dynamics of phages and bacteria in the poorly studied anaerobic environment (gut). In this study, therefore, we performed a comparative analysis to gain insights into the intestinal environment during COVID-19 infection by examining the fluctuations in the gut microbiota and DNA virome in patients with SARS-CoV-2 and healthy individuals.

## RESULTS

### Information on healthy participants and patients with COVID-19

In this study, bacterial identification was based on the 16S ribosomal RNA (rRNA) genome, and DNA virome analyses were performed using feces from 19 patients infected with SARS-CoV-2 (Table 1). All analyzed specimens were collected from patients infected with conventional SARS-CoV-2 strains (Wuhan and Wuhan/D614G). The patient cohort included 16 males and three females with a median age and body mass index (BMI, kg/m^2^) of 40 years and 23.8, respectively. Six of the 19 patients were taking antibiotics on admission, and six were on bowel regimens (Table S1). The healthy stool specimens used in the comparative analysis were obtained from 19 men with a median age of 46 years. All patients recovered and were discharged.

**TABLE 1:** Clinical background and characteristics of participants

Description	Cases (*n* = 19)	Healthy (*n* = 19)
Age: median, (IQR; interquartile range)	40 (32.5–48)	46 (35.5–56)
Gender male: n (ratio %)	16 (84.2%)	19 (100.0%)
BMI (body mass index: kg/m^2^) median, (IQR)	23.8 (22.5–26.5)	-
Antibiotics: n (ratio %)	6 (31.6%)	0 (0%)
Probiotics: n (ratio %)	6 (31.6%)	-
Symptoms during hospitalization: n (ratio %)		
Fever (≥37.0°C)	14 (73.7%)	-
Respiratory symptoms: n (ratio %)		
Cough	11 (57.9%)	-
Sore throat	6 (31.6%)	-
Dyspnea	5 (26.3%)	-
Diarrhea: n (ratio %)	5 (26.3%)	-
Chest CT (computed tomography) image: n (ratio %)		
Pneumonia	11 (57.9%)	-
Severity		
Mild	7 (36.8%)	-
Moderate	11 (57.9%)	-
Severe	1 (5.2%)	-

### Diversity and morphology analysis for the intestinal DNA virome

**Fig 1:**
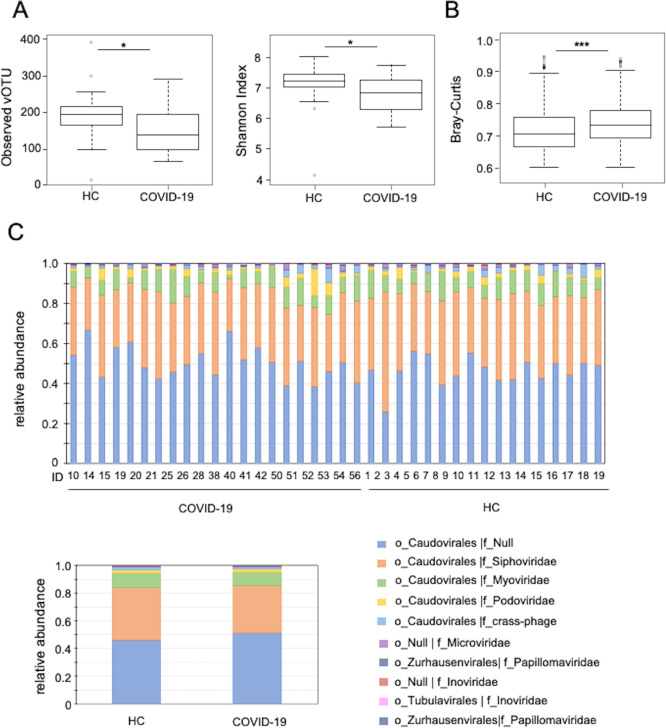
Changes in intestinal DNA virome diversity after COVID-19 onset (**A and B**), observed operational taxonomic units (OTUs) (left), Shannon analysis (right) (**B**), and Bray–Curtis dissimilarity to healthy samples. (**C**) Intestinal DNA virome compositions of the study participants (top). The mean intestinal DNA virome composition of healthy subjects and COVID-19 patients (bottom); o_” indicates an order, “f_” indicates a family-level classification, and "null" indicates unclassified. HC: healthy controls. *P < 0.05; ***P < 0.001 (Wilcoxon rank sum test).

First, a comparative analysis of the diversity of intestinal DNA virome was conducted between patients with COVID-19 and healthy controls. Samples from patients with COVID-19 were collected at the earliest point from the onset of COVID-19. Notably, viral vOTUs and the Shannon index (alpha diversity) were significantly reduced in samples from patients with COVID-19 than those from healthy controls (Fig. 1A). Bray–Curtis dissimilarity analysis, which measures diversity differences in samples, showed a significant difference in the diversity of intestinal DNA virome between patients with COVID-19 and healthy controls (Fig. 1B). Furthermore, we analyzed the fecal virome of 19 healthy individuals and 19 patients with COVID-19 to analyze the virome changes associated with gut microbiota changes after SARS-CoV-2 infection. The virome analysis pipeline is shown in Fig. S1. Each sample’s relative abundance was calculated using the sequence reads. DNA sequences that matched viral sequences in the MGV catalog were merged with MGV catalog metadata to classify viruses at order and family levels. This classification process assigned virus families based on the ICTV database and the crAss-like phage data. For unclassified viruses, order-level classification was performed using hidden Markov models in the Virus Orthologous Group database (<http://vogdb.org/>) (29). The composition of the gut DNA viral profile for each of the 19 patients with COVID-19 and 19 healthy participants revealed that the order Caudovirales, a double-stranded DNA phage with a tail, made up >95% of all participants (Fig. 1C top). At the family level, Siphoviridae was the most abundant in all participants, with individual differences in the proportions of Myoviridae and Podoviridae. In contrast, Caudovirales phages, which were not classified at the family level, accounted for approximately 50% of the participants. The intestinal DNA virome composition of patients with COVID-19 and healthy individuals was almost identical, with the groups showing no statistically significant differences (Fig. 1C bottom).

### Classification of phages based on host bacteria

**Fig 2:**
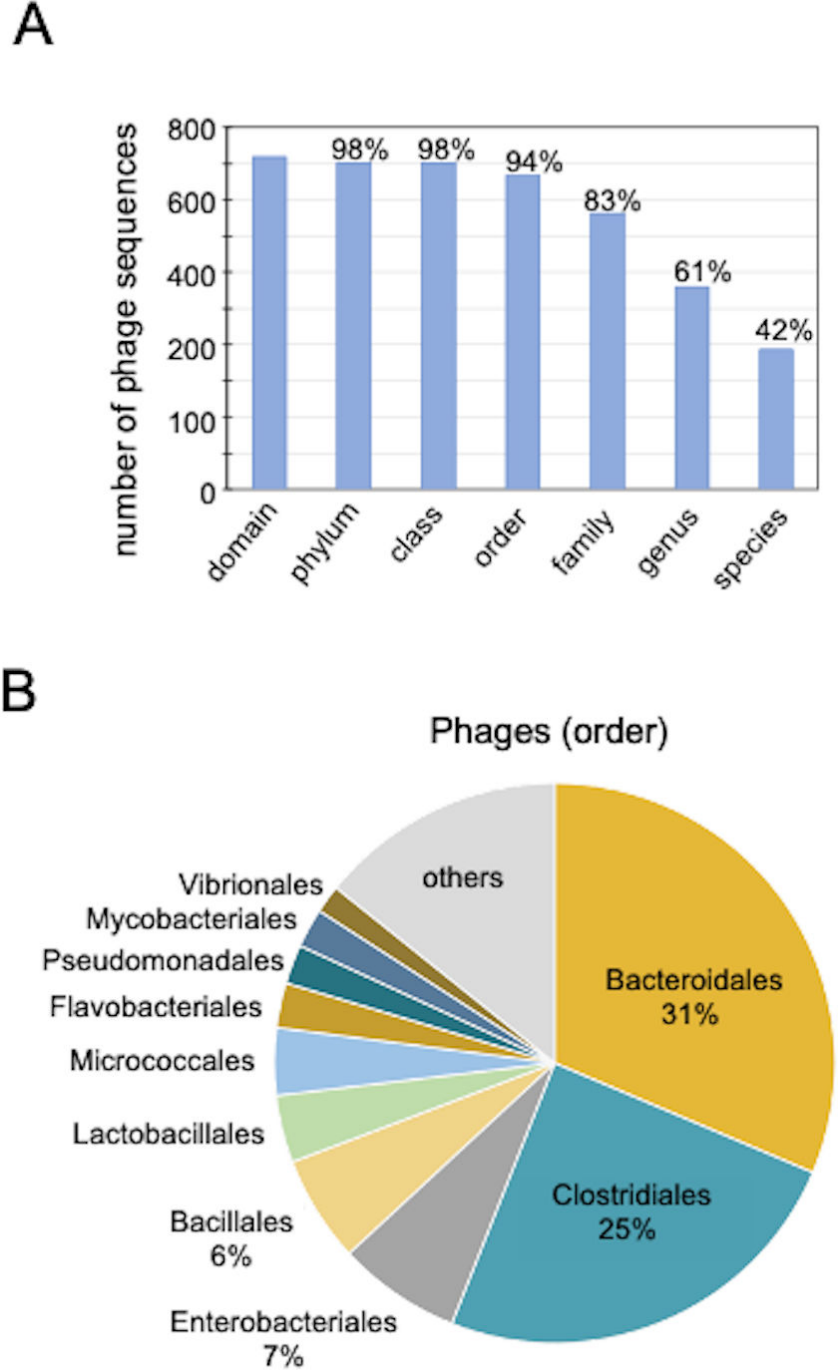
Classification of intestinal DNA virome based on host bacteria (**A**) Percentage of each hierarchical level in which all viral DNA (phage) sequences identified in the MGV catalog could be classified into their host bacteria. (**B**) Pie chart showing the top 10 host bacteria (order level) classified using the above method in healthy individuals.

The classification of the DNA virome based on morphology revealed that most intestinal DNA viruses are phages. Therefore, to investigate the interaction between intestinal phages and gut bacteria in their dynamics, all 51,272 viral sequences in the MGV catalog were integrated with the viral host data in the MGV catalog to identify the phage’s host bacteria. In total, 45,969 phage sequences (90%) provided host information, of which > 80% provided information down to the family level and >60% to the genus level (Fig. 2A). The classification of DNA viruses revealed several phages in healthy individuals in the order level: Bacteroidales, Clostridiales, Enterobacterales, and Bacillales (Fig. 2B).

### Comparative analysis of gut microbiota and phages in patients with SARS-CoV-2 and healthy controls

**Fig 3:**
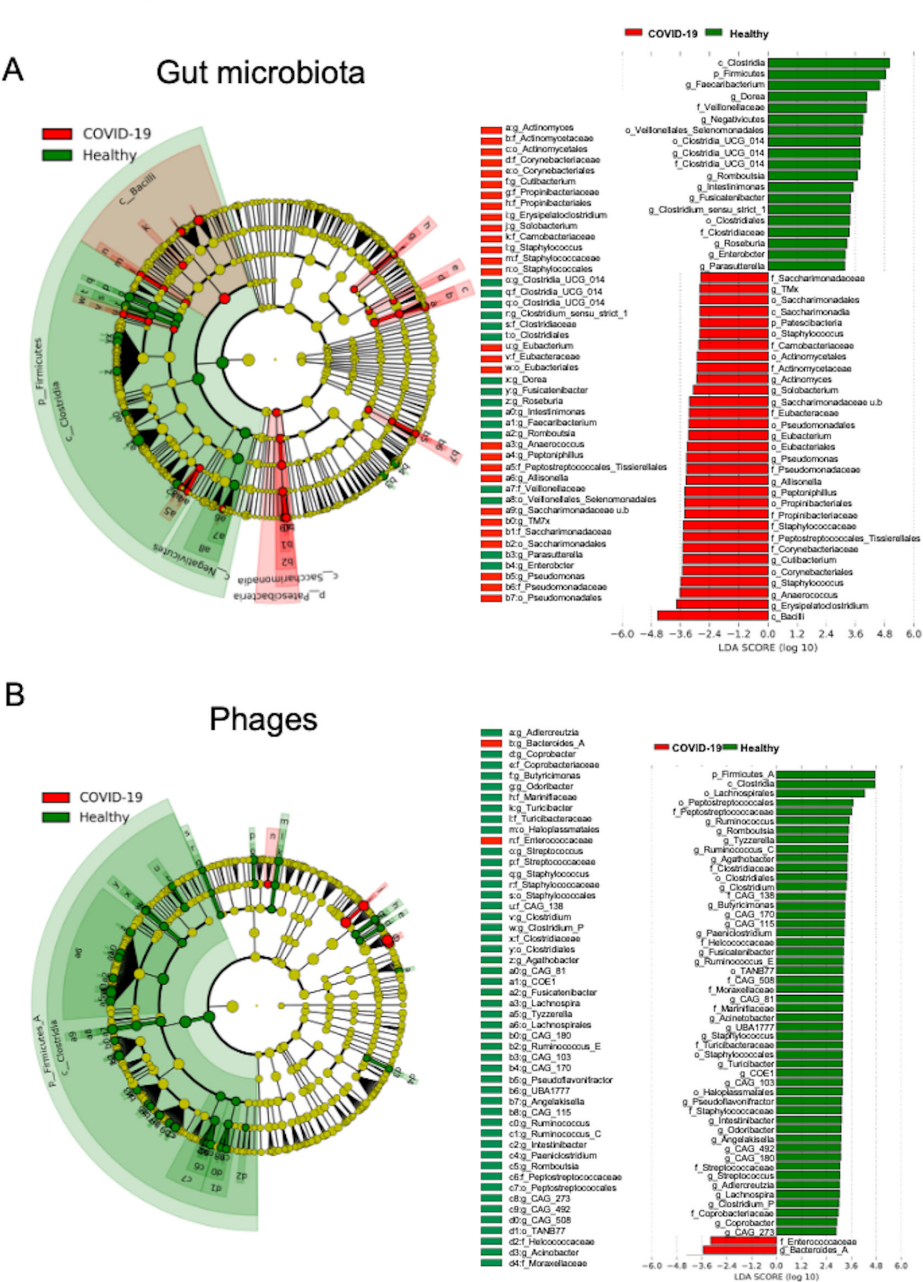
Comparative analysis of gut microbiota and phage in COVID-19 patients and healthy subjects. The earliest stool sample from the onset of disease was used from each of the 19 patients (average 8.1 days from the onset), one point per person, and compared with samples from 19 healthy subjects. Gut microbiota (**A**) and intestinal phage (**B**) were analyzed using linear discriminant analysis effect size (LEfSe). Effect size was LDA > 2.5

We compared the gut microbiota and intestinal phages of healthy individuals and patients with SARS-CoV-2 (Fig. 3). The condition for the analysis was to use the first specimens from individuals with SARS-CoV-2 after the onset of COVID-19 (one specimen per person). The median specimen collection date was 8.1 days (interval 3–18) from the onset of the illness. The linear discriminant analysis effect size (LEfSe) analysis was used to compare the groups’ gut microbiota profiles (Fig. 3A). Patients with COVID-19 showed a lower abundance ratio of bacteria belonging mainly to the Clostridia and Negativicutes class (phylum Firmicutes) than healthy controls. At the genus level, the abundance of *Clostridium_sensu_stricto_1*, *Dorea*, *Fusicatenibacter*, *Roseburia*, *Intestinimonas*, *Faecalibacterium*, and *Romboutsia* was significantly lower (Fig. 3A). In addition, bacteria belonging to the phylum Bacilli (*Erysipelatoclostridium*, *Staphylococcus*, and *Solobacterium* at the genus level), Saccharimonadaceae belonging bacteria, *Cutibacterium*, *Pseudomonas*, and *Actinomyces* were increased in patients with COVID-19 (Fig. 3A). Furthermore, comparison of intestinal phages showed that many phages infecting bacterial genera belonging to the class Clostridia decreased significantly in patients with COVID-19 compared with healthy controls. However, only phages infecting Enterococcaceae and *Bacteroides_A* were relatively increased (Fig. 3B).

### Comparative analysis of relative amounts of intestinal bacteria and phage after COVID-19 onset

**Fig 4:**
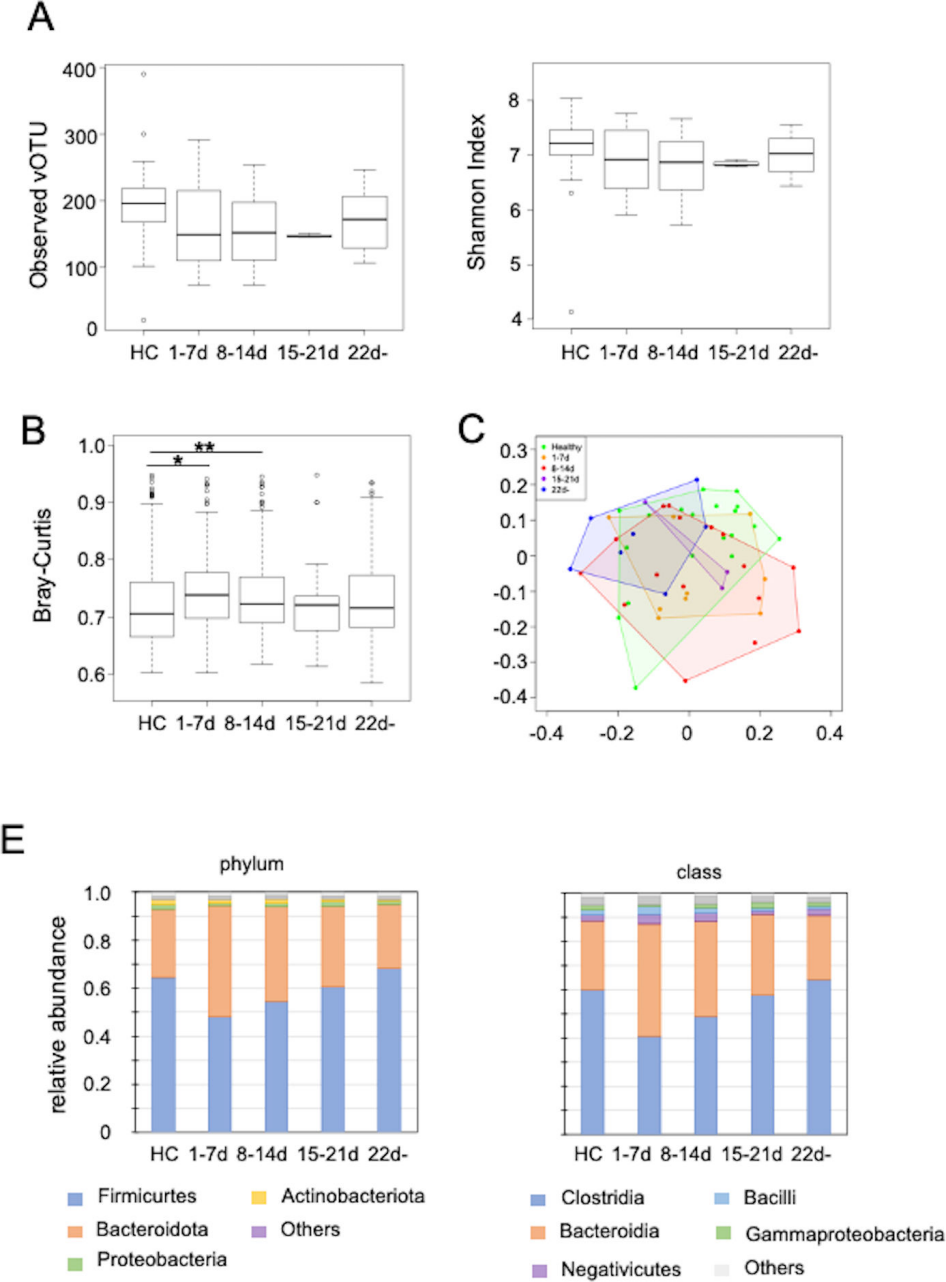
Temporal changes in intestinal DNA virome diversity after COVID-19 onset (**A and B**), observed operational taxonomic units (OTUs) (left), Shannon analysis (right) (**B**), and Bray–Curtis dissimilarity with healthy samples. (**C**) Two-dimensional plot of changes in Bray–Curtis dissimilarity over time (PCoA plot). (**D**) Intestinal phages classified by host bacteria at the phylum (left) and class (right) levels from COVID-19 onset. HC; healthy control, 1–7d: within 7 days of COVID-19 onset, 8–14d: 8–14 days after COVID-19 onset, 15–21d: 15–21 days after COVID-19 onset, and 22d-: over 22 days after COVID-19 onset **P < 0.01, ***P < 0.001 (Wilcoxon rank sum test)

We next examined the relative amounts of intestinal phages from the onset of COVID-19. First, samples from patients with COVID-19 were divided into four categories based on the number of days from the onset date of COVID-19: within 7 days (nine samples), 8–14 days (16 samples), 15–21 days (three samples), and after 22 days (seven samples) (Table S1). The number of samples collected within 15–21 days was relatively small because most patients were discharged from the hospital within approximately 14 days. After discharge from the hospital, several patients visited the outpatient clinic (3 weeks after onset) and provided stool specimens. The observed vOTUs and Shannon index results indicated no statistically significant differences compared with those of healthy individuals. However, both measures decreased within 7 days after onset, followed by a slight increase after day 22 (Fig. 4A). β-diversity analysis using Bray–Curtis dissimilarity revealed statistically significant differences between patients with COVID-19 and healthy controls on days 1–7 and 8–14 (Fig. 4B). A two-dimensional plot was generated using principal coordinate analysis (PCoA) with a distance matrix of Bray–Curtis dissimilarities. (Fig. 4C). The sample of patients with COVID-19 was color-coded in four periods, showing that all clusters overlapped in part with healthy participants, with the samples from days 1 to 7 and 8 to 14 being more widely scattered. The relative abundance of phages after COVID-19 onset was the most different from that of healthy individuals within 7 days after onset at the phylum and class levels (Fig. 4D).

### Differences in relative amounts of intestinal bacteria and phage after COVID-19 onset

**Fig 5:**
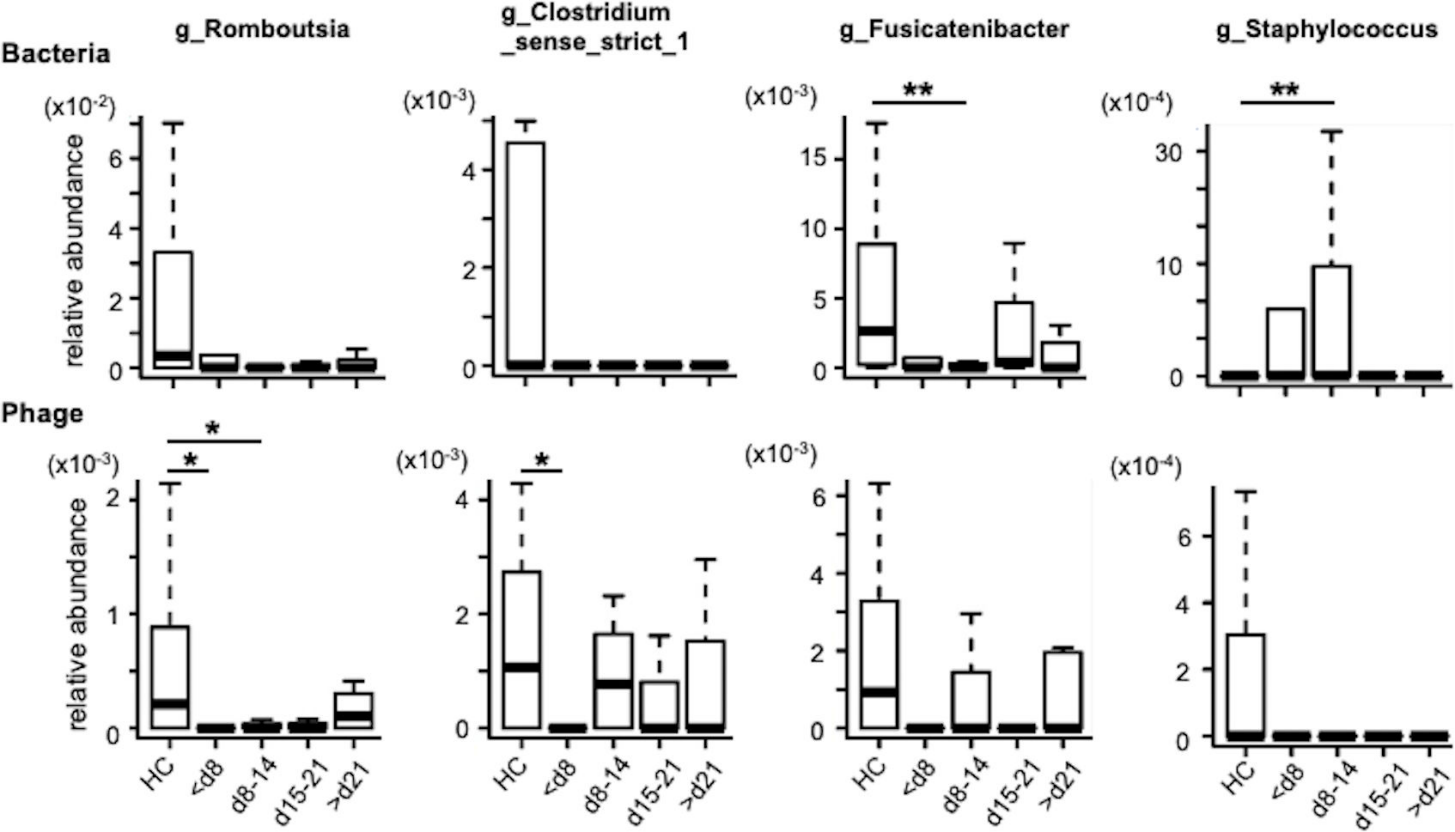
The relative abundance of intestinal phage and their host bacteria over time since the onset of COVID-19. Gut microbiota (top) and their phages (bottom) 1–7d: within 7 days of COVID-19 onset, 8–14d: 8–14 days after COVID-19 onset, 15–21d: 15–21 days after COVID-19 onset, 22d-: over 22 days after COVID-19 onset, *P < 0.05; **P < 0.01 (Dunn’s multiple comparisons test).

We analyzed the correlation between the differences in the relative amounts of gut microbiota and the intestinal phages hosting these bacteria after the onset of COVID-19. This analysis focused on gut microbiota, for which changes were observed after the onset of COVID-19. Changes over time in the relative abundance of phages infecting *Romboutsia*, *Clostridium*, *Fusicatenibacter,* and *Staphylococcus* are shown in the top panel in Fig. 5. Notably, the four phage species showed decreased abundance relative to healthy controls on days 1–7 of COVID-19 onset. Furthermore, the relative abundance of the three phages, except for the staphylococcal phage, showed a recovery trend to varying degrees, compared to the early stages of the disease. However, a consistent decrease was observed for the staphylococcal phage at all time points.

While the relative abundances of *Romboutsia*, *Clostridium_sense_strict_1*, and *Fusicatenibacter* were reduced compared with those in healthy controls on days 1–7 of COVID-19 onset (Fig. 5, bottom), no recovery of *Romboutsia* or *Clostridium_sense_strict_1* was observed during the study. The recovery of *Fusicatenibacter* was observed after the third week of onset, but its relative abundance tended to be suppressed after the second week when the *Fusicatenibacter* phage was detected. In contrast, a transient increase in *Staphylococcus* was observed immediately after the onset of COVID-19 compared with healthy controls during the first 2 weeks.

### Comparative analysis of virulent and temperate scores in VLPs between patients with COVID-19 and healthy controls

**Fig 6:**
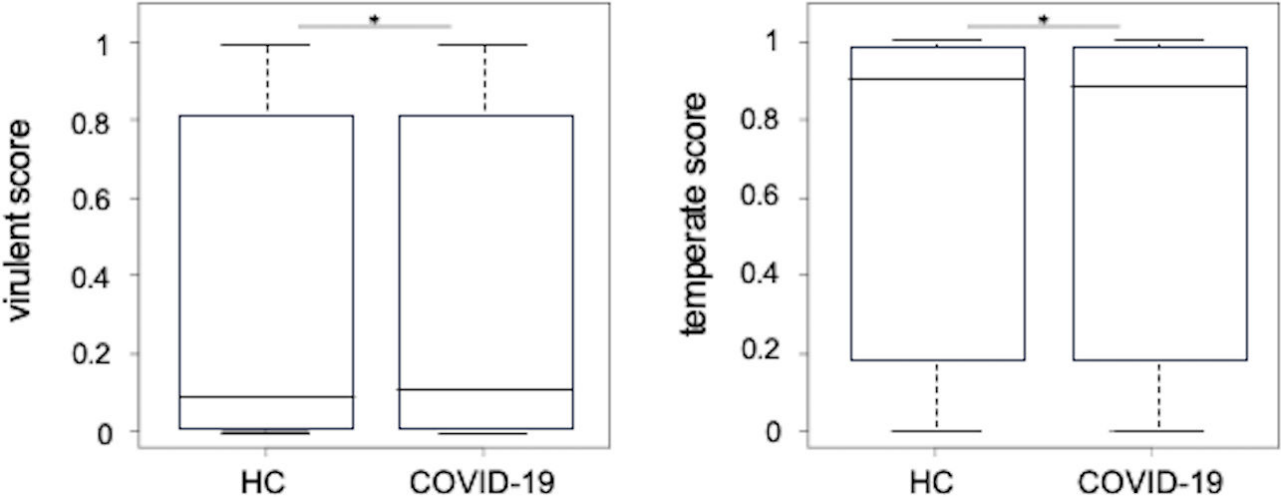
The proportion of virulent phage and temperate phages present in VLPs. Comparative analysis of the virulent (left) and temperate (right) scores of phage sequences detected in COVID-19 and healthy groups. *P < 0.05 (Wilcoxon rank sum test).

Based on their lifecycle, phages are classified into virulent (lytic phages) and temperate phages (lysogenic phages) (11). While acting as a prophage, the host bacterium replicates the phage genome during cell division. Prophages shift through the lysogenic cycle when triggered by environmental signals such as the bacterial SOS response. Therefore, we analyzed the effect of the life cycle on phages due to the onset of COVID-19. The MGV catalog database was consulted, and the identified viral sequences were integrated with the MGV catalog metadata to obtain virulent and temperate scores for phages. The virulent and temperate scores indicate the probabilities of being virulent (lytic phage) and temperate phages (lysogenic phage), respectively (31). Therefore, to determine whether there was a difference in the proportion of virulent and temperate phages in viral fractions (VLPs) between patients with COVID-19 and healthy controls, we performed a comparative analysis of the virulent and temperate scores of phage sequences detected in both groups (Fig. 6). Basically, both groups tended to have lower virulent scores and higher temperate scores. We observed a significant increase in the virulent score (Fig. 6, left) and a significant decrease in the temperate score (Fig. 6, right) after the onset of COVID-19.

## DISCUSSION

This study aimed to investigate the interrelated behaviors between the intestinal DNA viral community and gut microbiota using SARS-CoV-2 infection as a model. First, we categorized enteric DNA viruses (enteric phages) into orders and families by assessing phage morphology. Subsequently, enteric phages were classified based on the host bacteria. We used the time-series analysis of 16S rRNA gut microbiota and free phages from the same samples to uncover the dynamic relationships between Enterobacteria and their phages during the transient period following the onset of COVID-19. This study provides insights into the dynamic interactions between the enteric DNA virus community and gut microbiota from the inception of infectious disease onset.

Previous studies have shown the varying composition of intestinal phages in healthy individuals in Japan (20). Similarly, the analysis of the intestinal DNA viral profile based on the ICTV database revealed differences in the proportions of Myoviridae among individuals (32). In this study, patients with COVID-19 and healthy controls showed no differences in the composition, which is consistent with previous reports (33). However, there is a report that the relative abundance of crAss-like phages decreased in patients with COVID-19 compared with healthy controls, possibly due to the different databases used for the analysis (34). The classification of phages based on the host bacteria showed that patients with COVID-19 had a lower proportion of phages that infect bacteria, primarily belonging to the phylum Firmicutes/Clostridia, with this being particularly noticeable on days 1–7 after the onset of COVID-19. This finding suggests that gut bacterial and viral profile changes occur early in the progression of COVID-19, which is consistent with the findings of previous studies and the current study’s diversity analysis and intergroup comparison of intestinal phage classification by host bacteria (35).

Intestinal phages and bacteria have a parasite–host relationship. Therefore, instead of classification based on the phage morphology, analysis of the dynamics of the individual corresponding intestinal phages and host bacteria provides a glimpse into their offensive and defensive activities in the intestinal environment. Overall, after COVID-19 onset, Clostridia class members decreased in COVID-19 patients (6–8), and a marked decrease in the number of free phages that hosted them was observed immediately after the onset of COVID-19. These bacteria are considered obligate anaerobes, and their central metabolic pathway is inhibited by elevated oxygen levels (36, 37). This suggests that a lack of short-chain fatty acids may negatively affect the intestinal epithelium immediately after COVID-19 onset, leading to increased oxygen levels in the intestinal tract. It is known that phage infection depends on the metabolic state of the host bacteria (38). When host bacteria are under stress conditions such as starvation, phage genomes injected into the bacteria are maintained as episomes (pseudo-lysogenic phages) and the lysogenic cycle is halted until environmental conditions improve (15, 39). As observed in *Romboutsia*, the phages were unable to proceed through the lytic cycle (DNA replication, synthesis of progeny phages, particle assembly, lysis, and release) and remained in the bacteria as pseudo-lytic phages, which may have led to a decrease in free phages. While the fact that the percentage of free phages increased before the host bacteria, as observed in *Clostridium*, suggests that the release of free phages by lytic infection may have hindered the host bacteria from multiplying, interestingly, the relative abundances of *Fusicatenibacter* and their phages after the onset of COVID-19 appeared to be mutually exclusive. In addition, immediately after the onset of COVID-19, increase in facultative anaerobes such as *Staphylococcus* was observed (40), and decreased phages infecting them were observed. A phage life cycle switching associated with environmental changes can be assumed for facultative anaerobic bacteriophages since changes in metabolic pathways between aerobic and anaerobic environments have been reported for facultative anaerobic bacteria (38). The increased relative abundance of *Staphylococcus* observed immediately after the onset of COVID-19 suggests a molecular mechanism that suppresses the lytic ability of *Staphylococcus* phages, although the details remain unknown. These observations indicate that a conflicting aspect between the phage and its host bacteria is reflected in the intestinal environment.

These observations may be referenced to the previously proposed phage selection models for lytic and lysogenic switching. The “Piggyback-the-Winner” model predicts that when microorganism abundance and growth rates are high in environments favorable to bacteria (high nutrient levels), phages are incorporated into the host genome as prophages (some phages replicate as plasmids) and replicate their DNA as the host bacteria divide (41, 42). Conversely, phages promote lysogenic activity in environments with reduced bacterial density (41). Notably, phages (mainly tailed double-stranded DNA bacteriophage particles) can interact directly with mucins by expressing proteins with immunoglobulin-like domains on the capsid, termed bacteriophage mucus attachments, which are phage-mediated defense systems on intestinal mucosal epithelia (43). These are generally models in which the formation of a spatial gradient of lysis and lysogenicity on the mucosal surface of the intestinal mucosal epithelium–basal mucin, phage-dominated middle, and bacterial flora-dominated upper layers acts as a multiple defense strategy against pathogens (43). In the present analysis, the subjects generally tended to have higher temperate scores than virulence scores in the prediction of phage types, consistent with reports that most human intestinal phages are temperate type (44). However, considering the increase in virulence score and decrease in temperate score after the onset of COVID-19, it is possible that transient immune activation of the mucosal epithelium in COVID-19 triggered dynamic changes in phages and their host bacteria. That is, the phage and host bacterial dynamics observed in this study appear to be consistent with the “Piggyback-the-Winner” model, in which phages select for the lysogenic cycle in environments where host bacteria are depleted and for lysogenization in environments where host bacteria are increasing in number.

This study has some limitations. First, it only focused on free intestinal DNA viruses and did not analyze phages present in bacteria, such as prophages incorporated into the bacterial genome. Second, the study did not examine the direct relationship between intestinal DNA virome and the pathogenesis and severity of COVID-19. Lastly, it has been reported that phages directly affect host immunity (42, 45) and antibiotics affect the intestinal DNA virome (34, 46); however, the present analysis did not include the effects of medication or steroid use on the intestinal DNA virome. The potential impact of vaccination on the DNA virome is a subject that could be explored in future research, as it has been suggested that the symbiotic bacteria may be affected by vaccination (47). The cohort size in this study (*n* = 19 subjects per group) was relatively small. The number and timing of stool samples differed in this analysis, making it impossible to perform a comparative analysis of the time-series data from the same subjects. In particular, the number of analysis groups (*n* = 3) of samples from 15 to 21 days after COVID-19 onset was small; therefore, caution should be exercised when interpreting the results.

In conclusion, the present study reports correlated movements reminiscent of the survival strategy unfolding between the intestinal DNA virome (intestinal phageome) and the gut microbiota immediately after the onset of disease in COVID-19 patients. Since the host range of phages is narrow and host bacteria are generally restricted to the species or strain level (16), the analysis of gut microbiota at the species level will lead to a more detailed understanding of the relationship between intestinal bacteria and phages. This study demonstrated an example of mutual regulation between some intestinal phages and bacteria in COVID-19 pathogenesis and clarified some intestinal environmental changes. Further studies on the relationship between the intestinal DNA virome and gut microbiota, including prophages and pseudo-lysogenic phages, are needed in the context of intestinal environmental changes. Through increased observative or extensive molecular biological studies, our understanding of the molecular basis of the lytic and lysogenic cycles and their host dependency in bacteriophages can be improved. This knowledge will not only benefit academia but will also lead to practical applications for regulating gut microbiota.

## MATERIALS AND METHODS

### Subject recruitment and sample collection

Stool samples were collected from 19 COVID-19 patients who were hospitalized at the University of Tokyo Institute of Medical Science from March to August 2020 (Table 1). In Japan, at that time, all symptomatic COVID-19 patients required hospitalization until two viral PCR tests were confirmed to be negative. Patients diagnosed with COVID-19 were categorized into three groups based on a prior report (27). Those who experienced various symptoms, such as fever, cough, and sore throat, but did not have difficulty breathing or CT images indicating pneumonia were classified as having a mild form of the disease. Individuals with CT images showing pneumonia and exhibiting symptoms such as fever, respiratory issues, and an oxygen saturation level ≥94% were classified as moderate cases. Finally, those with CT images displaying pneumonia and an oxygen saturation level below 94% were classified as severe cases. Patients’ stool samples were collected upon admission and discharge, and some discharged patients provided stool samples on follow-up days. After collection, stool samples were stored at −80°C until DNA extraction. As a control group, 19 healthy individuals age-matched to SARS-CoV-2-infected patients were randomly recruited to participate in the study. Those who had taken antibiotics within the past 2 weeks were excluded. The stool specimens were provided by each of them and subjected to the same analysis.

### Isolation of free viral and bacterial fractions from stool specimens

Frozen stool specimens (1 g) were thawed and suspended in 3 mL of SM-plus Buffer (100 mM NaCl, 50 mM Tris-HCl [pH 7.4], 8 mM MgSO4, 5 mM CaCl2, 0.01% [wt/vol] gelatin) with a vortex mixer. The suspension was centrifuged (6,000 x *g*, 5 minutes), and the supernatant was collected in a new tube. This procedure was repeated twice, and the combined supernatant was further centrifuged (6,000 × *g*, 15 minutes). The supernatant was collected again and filtered through a 0.45-µm filtration membrane and used as the viral fraction (VLPs). To the pellet obtained by centrifugation, 5 mL of SM-plus Buffer was added and suspended with a vortex mixer, then filtered through a 100-µm cell strainer (Corning, Inc., Corning, NY, USA), and the filtered bacterial suspension was used as the bacterial fraction. DNA was extracted from the viral and bacterial fractions.

### DNA extraction, amplification, and gene sequencing

DNA was extracted from viral and bacterial fractions derived from fecal samples as previously described. A total of 41 16S rRNA gene libraries were prepared according to the 16S Metagenomics Sequencing Library Preparation Guide (Illumina, San Diego, CA, USA, Part #15044223 Rev. B). The hypervariable V3–V4 region of the 16S rRNA gene was then amplified. Sequencing was performed with an Illumina MiSeq (Illumina) using the MiSeq Reagent Kit v3 (600 cycles) with a 20% PhiX (Illumina) spike-in.

For viral DNA sequencing, gene libraries were created using the Qiagen Fx DNA library kit (Qiagen, Hilden, Germany), and shot-gun analysis was performed with NovaSeq 6000 using NovaSeq 6000 S2 Reagent Kit v1.5 (300 cycles).

### Pipeline for 16s rRNA data analytics

Bacterial 16S sequences were filtered for quality, denoised, and analyzed using Quantitative Insights Into Microbial Ecology 2 (QIIME 2) (48). Briefly, paired-end reads were denoised to amplicon sequence variants using DADA2 (49). Bacterial classification was assigned to the resulting amplicon sequence variants based on the SILVA database (release 132) (50). This region was trimmed to the V3–V4 region of the 16S rRNA gene using a naïve Bayesian classification method (51).

### Pipeline and procedure for the analysis of intestinal viral DNA

The total amount of sequencing data obtained by NovaSeq 6000 sequencing was 152.3 Gb for 35 samples from COVID-19 patients and 19 samples from healthy subjects (total 54 samples), with 1,114,944,944 paired-end reads (median 16,649,207, quartile range 11,364,448–23,200,779). Sequence reads were trimmed, and adapter sequences were removed using Trimmomatic (52) (v0.39) (ILLUMINACLIP: adapters/TruSeq3-PE-2. fa:2:30:10 LEADING:20 TRAILING:20 MINLEN:75). Duplicate reads with exact matches were removed using PRINSEQ (53) (v0.20.4). The trimmed reads were evaluated using FastQC (v0.11.9) to confirm the improved quality. *De novo* assembly was then performed using metaSPAdes (54) (v3.15.3). Scaffolding genome assembly for the predicted viral genome was performed using SeqKit (55) (v2.0.0) (seq -m 1000) to remove scaffolds of less than 1 kb. The number of scaffolds larger than 1 kb was 237, 589 (median 3,763, interquartile range, 1,592–5,704) for a total of 56 samples. These 1-kb or larger scaffolds were used for viral sequence exploration and classification.

A combination of five methods was used to search for viral sequences to prevent false negatives. Scaffolds meeting any of the following five conditions were extracted as viral sequences (1). Blastn alignment (56) (v2.9.0+) (evalue 1e-10) against NCBI nucleotide (nt) collection resulted in the best hit with the lowest e-value being the virus (2), contigs (https:// github.com/Microbiology/ccontigs) (v1.0.0) (-l 50 t 1.0), which was found to be a cyclic genome (3); VIBRANT (57) (v1.2.1) (-virome) hit (excluding provirus) (4), VirSorter2 (58) (v2.2.3) (excluding proviruses), and (5) hits on VirFinder (59) (v1.1) based on the Metagenomic Gut Virus (MGV) viral sequence search pipeline (29), which was determined to be a viral sequence. The number of viral sequences was 52,324 (median 842; interquartile range 485–1,285) for 56 samples. To resolve the effects of individual differences, it is necessary to cluster viral sequences to higher taxonomic levels, such as genera and families (60). Therefore, before conducting the diversity analysis of the intestinal DNA virome, we clustered all 52,324 viral sequences to the family level using the Average Amino Acid Identity (AAI) method combined with gene sharing (29), yielding 6,801 vOTUs (viral operational taxonomic units (vOTUs). Data dilution based on coverage (61) was also performed to account for differences in the number of sequences among the samples.

Next, the MGV catalog (29), an enteric DNA virus database, was used to classify the viral sequences as follows: Blastn alignment (56) (v2.9.0+) (-evalue 1e-10) and Blastx alignment (56) (v2). The total number of hits in the MGV catalog was 51,272 (median 827; interquartile range; 476–1,267) for 56 samples, and approximately 98% of the viral sequences were homologous to sequences in the MGV catalog. The MGV genome numbers were then merged with the MGV catalog metadata to obtain the viral taxonomy name by the International Committee on Taxonomy of Viruses and the Viral Temperate Score of the phage for each viral sequence. In addition, the MGV genome numbers were integrated with viral host data to determine the host (primarily the host bacterium) for each viral sequence. The relative abundance of virus (phages) was calculated by dividing the number of host bacteria for each identified viral sequence by the total number.

The information for each sequence sample is listed in Table S2. Despite variations in the count of analysis reads between the healthy control and COVID-19 patient groups, no differences were observed between the two groups in terms of the number of identified viral sequences or the number of sequences that matched the MGV catalog (Fig. S2). Based on these findings, we determined that it was valid to compare the ratios of viral sequences obtained between the groups.

### Statistical analysis

The R package (v3.6.2) and vegan package (v2.5–7) (<https://cran.r-project.org/web/packages/vegan/>) were used for analyzing the diversity of the intestinal DNA virome. To evaluate the alpha diversity, the Shannon and Bray–Curtis indices were used. Principal coordinate analysis (PCoA) was used to enhance the visualization of beta diversity. The Wilcoxon rank-sum test (Mann–Whitney comparisons test) was used to compare each group of COVID-19 patients and healthy subjects. Additionally, linear discriminant analysis effect size (LEfSe) analysis was performed with a threshold for log LDA score of 2.5 and a *P* value threshold of 0.05, to identify significant differences between the groups.

## Data Availability

Data described in this study are openly available in DNA Data Bank of Japan (DDBJ) (https://ddbj.nig.ac.jp/search; accession number: PRJDB12349 and PRJDB18646).

## SUPPLEMENTAL MATERIAL

Supplemental figures spectrum.00998-24-s0001.pdf Fig. S1 and S2.

Supplemental tables spectrum.00998-24-s0002.xlsx Tables S1 and S2.

ASM does not own the copyrights to Supplemental Material that may be linked to, or accessed through, an article. The authors have granted ASM a non-exclusive, world-wide license to publish the Supplemental Material files. Please contact the corresponding author directly for reuse.

## References

[1] Al Maqbali M, Al Badi K, Al Sinani M, Madkhali N, Dickens GL. 2022. Clinical features of COVID-19 patients in the first year of pandemic: a systematic review and meta-analysis. Biol Res Nurs 24:172–185. doi:10.1177/10998004211055866 34866409 PMC8968436

[2] Gaber Y. 2020. Diarrhoea and the COVID-19 pandemic. Arab J Gastroenterol 21:146–150. doi:10.1016/j.ajg.2020.06.001 32680695 PMC7833714

[3] Jiehao C, Jin X, Daojiong L, Zhi Y, Lei X, Zhenghai Q, Yuehua Z, Hua Z, Ran J, Pengcheng L, Xiangshi W, Yanling G, Aimei X, He T, Hailing C, Chuning W, Jingjing L, Jianshe W, Mei Z. 2020. A case series of children with 2019 novel coronavirus infection: clinical and epidemiological features. Clin Infect Dis 71:1547–1551. doi:10.1093/cid/ciaa198 32112072 PMC7108143

[4] Vijay A, Valdes AM. 2022. Role of the gut microbiome in chronic diseases: a narrative review. Eur J Clin Nutr 76:489–501. doi:10.1038/s41430-021-00991-6 34584224 PMC8477631

[5] Mizutani T, Ishizaka A, Koga M, Tsutsumi T, Yotsuyanagi H. 2022. Role of microbiota in viral infections and pathological progression. Viruses 14:950. doi:10.3390/v14050950 35632692 PMC9144409

[6] Mizutani T, Ishizaka A, Koga M, Ikeuchi K, Saito M, Adachi E, Yamayoshi S, Iwatsuki-Horimoto K, Yasuhara A, Kiyono H, Matano T, Suzuki Y, Tsutsumi T, Kawaoka Y, Yotsuyanagi H. 2022. Correlation analysis between gut microbiota alterations and the cytokine response in patients with coronavirus disease during hospitalization. Microbiol Spectr 10:e0168921. doi:10.1128/spectrum.01689-21 35254122 PMC9045125

[7] Yeoh YK, Zuo T, Lui G-Y, Zhang F, Liu Q, Li AY, Chung AC, Cheung CP, Tso EY, Fung KS, Chan V, Ling L, Joynt G, Hui D-C, Chow KM, Ng SSS, Li T-M, Ng RW, Yip TC, Wong G-H, Chan FK, Wong CK, Chan PK, Ng SC. 2021. Gut microbiota composition reflects disease severity and dysfunctional immune responses in patients with COVID-19. Gut 70:698–706. doi:10.1136/gutjnl-2020-323020 33431578 PMC7804842

[8] Zuo T, Zhang F, Lui GCY, Yeoh YK, Li AYL, Zhan H, Wan Y, Chung ACK, Cheung CP, Chen N, Lai CKC, Chen Z, Tso EYK, Fung KSC, Chan V, Ling L, Joynt G, Hui DSC, Chan FKL, Chan PKS, Ng SC. 2020. Alterations in gut microbiota of patients with COVID-19 during time of hospitalization. Gastroenterology 159:944–955. doi:10.1053/j.gastro.2020.05.048 32442562 PMC7237927

[9] Ishizaka A, Koga M, Mizutani T, Yamayoshi S, Iwatsuki-Horimoto K, Adachi E, Suzuki Y, Kawaoka Y, Yotsuyanagi H. 2024. Association of gut microbiota with the pathogenesis of SARS-CoV-2 Infection in people living with HIV. BMC Microbiol 24:6. doi:10.1186/s12866-023-03157-5 38172680 PMC10763188

[10] Yamamoto S, Saito M, Tamura A, Prawisuda D, Mizutani T, Yotsuyanagi H. 2021. The human microbiome and COVID-19: a systematic review. PLoS One 16:e0253293. doi:10.1371/journal.pone.0253293 34161373 PMC8221462

[11] Gu S, Chen Y, Wu Z, Chen Y, Gao H, Lv L, Guo F, Zhang X, Luo R, Huang C, Lu H, Zheng B, Zhang J, Yan R, Zhang H, Jiang H, Xu Q, Guo J, Gong Y, Tang L, Li L. 2020. Alterations of the gut microbiota in patients with coronavirus disease 2019 or H1N1 influenza. Clin Infect Dis 71:2669–2678. doi:10.1093/cid/ciaa709 32497191 PMC7314193

[12] Zuo T, Wu X, Wen W, Lan P. 2021. Gut microbiome alterations in COVID-19. Genomics Proteomics Bioinformatics 19:679–688. doi:10.1016/j.gpb.2021.09.004 34560321 PMC8478109

[13] Liu Q, Mak JWY, Su Q, Yeoh YK, Lui G-Y, Ng SSS, Zhang F, Li AYL, Lu W, Hui D-C, Chan PK, Chan FKL, Ng SC. 2022. Gut microbiota dynamics in a prospective cohort of patients with post-acute COVID-19 syndrome. Gut 71:544–552. doi:10.1136/gutjnl-2021-325989 35082169

[14] Li Y, Handley SA, Baldridge MT. 2021. The dark side of the gut: virome-host interactions in intestinal homeostasis and disease. J Exp Med 218:e20201044. doi:10.1084/jem.20201044 33760921 PMC8006857

[15] Olszak T, Latka A, Roszniowski B, Valvano MA, Drulis-Kawa Z. 2017. Phage life cycles behind bacterial biodiversity. Curr Med Chem 24:3987–4001. doi:10.2174/0929867324666170413100136 28412903

[16] Díaz-Muñoz SL, Koskella B. 2014. Bacteria-phage interactions in natural environments. Adv Appl Microbiol 89:135–183. doi:10.1016/B978-0-12-800259-9.00004-4 25131402

[17] Brady A, Quiles-Puchalt N, Gallego Del Sol F, Zamora-Caballero S, Felipe-Ruíz A, Val-Calvo J, Meijer WJJ, Marina A, Penadés JR. 2021. The arbitrium system controls prophage induction. Curr Biol 31:5037–5045. doi:10.1016/j.cub.2021.08.072 34562384 PMC8612738

[18] Brady A, Felipe-Ruiz A, Gallego Del Sol F, Marina A, Quiles-Puchalt N, Penadés JR. 2021. Molecular basis of lysis-lysogeny decisions in Gram-positive phages. Annu Rev Microbiol 75:563–581. doi:10.1146/annurev-micro-033121-020757 34343015

[19] Bruce JB, Lion S, Buckling A, Westra ER, Gandon S. 2021. Regulation of prophage induction and lysogenization by phage communication systems. Curr Biol 31:5046–5051. doi:10.1016/j.cub.2021.08.073 34562385 PMC8612742

[20] Fujimoto K, Kimura Y, Shimohigoshi M, Satoh T, Sato S, Tremmel G, Uematsu M, Kawaguchi Y, Usui Y, Nakano Y, Hayashi T, Kashima K, Yuki Y, Yamaguchi K, Furukawa Y, Kakuta M, Akiyama Y, Yamaguchi R, Crowe SE, Ernst PB, Miyano S, Kiyono H, Imoto S, Uematsu S. 2020. Metagenome data on intestinal phage-bacteria associations aids the development of phage therapy against pathobionts. Cell Host Microbe 28:380–389. doi:10.1016/j.chom.2020.06.005 32652061

[21] Sausset R, Petit MA, Gaboriau-Routhiau V, De Paepe M. 2020. New insights into intestinal phages. Muc Immunol 13:205–215. doi:10.1038/s41385-019-0250-5 PMC7039812 31907364

[22] Norman JM, Handley SA, Baldridge MT, Droit L, Liu CY, Keller BC, Kambal A, Monaco CL, Zhao G, Fleshner P, Stappenbeck TS, McGovern DPB, Keshavarzian A, Mutlu EA, Sauk J, Gevers D, Xavier RJ, Wang D, Parkes M, Virgin HW. 2015. Disease-specific alterations in the enteric virome in inflammatory bowel disease. Cell 160:447–460. doi:10.1016/j.cell.2015.01.002 25619688 PMC4312520

[23] Chen Q, Ma X, Li C, Shen Y, Zhu W, Zhang Y, Guo X, Zhou J, Liu C. 2020. Enteric phageome alterations in patients with type 2 diabetes. Front Cell Infect Microbiol 10:575084. doi:10.3389/fcimb.2020.575084 33552999 PMC7862107

[24] Ma Y, You X, Mai G, Tokuyasu T, Liu C. 2018. A human gut phage catalog correlates the gut phageome with type 2 diabetes. Microbiome 6:24. doi:10.1186/s40168-018-0410-y 29391057 PMC5796561

[25] Kim M-S, Park E-J, Roh SW, Bae J-W. 2011. Diversity and abundance of single-stranded DNA viruses in human feces. Appl Environ Microbiol 77:8062–8070. doi:10.1128/AEM.06331-11 21948823 PMC3208976

[26] Fitzgerald CB, Shkoporov AN, Upadrasta A, Khokhlova EV, Ross RP, Hill C. 2021. Probing the "Dark matter" of the human gut phageome: culture assisted metagenomics enables rapid discovery and host-linking for novel bacteriophages. Front Cell Infect Microbiol 11:616918. doi:10.3389/fcimb.2021.616918 33791236 PMC8005731

[27] Neil JA, Cadwell K. 2018. The intestinal virome and immunity. J Immunol 201:1615–1624. doi:10.4049/jimmunol.1800631 30181300 PMC6179364

[28] Simmonds P, Aiewsakun P. 2018. Virus classification - where do you draw the line? Arch Virol 163:2037–2046. doi:10.1007/s00705-018-3938-z 30039318 PMC6096723

[29] Nayfach S, Páez-Espino D, Call L, Low SJ, Sberro H, Ivanova NN, Proal AD, Fischbach MA, Bhatt AS, Hugenholtz P, Kyrpides NC. 2021. Metagenomic compendium of 189,680 DNA viruses from the human gut microbiome. Nat Microbiol 6:960–970. doi:10.1038/s41564-021-00928-6 34168315 PMC8241571

[30] Roux S, Adriaenssens EM, Dutilh BE, Koonin EV, Kropinski AM, Krupovic M, Kuhn JH, Lavigne R, Brister JR, Varsani A, et al.. 2019. Minimum information about an uncultivated virus genome (MIUViG). Nat Biotechnol 37:29–37. doi:10.1038/nbt.4306 30556814 PMC6871006

[31] Hockenberry AJ, Wilke CO. 2021. BACPHLIP: predicting bacteriophage lifestyle from conserved protein domains. PeerJ 9:e11396. doi:10.7717/peerj.11396 33996289 PMC8106911

[32] Barylski J, Enault F, Dutilh BE, Schuller MB, Edwards RA, Gillis A, Klumpp J, Knezevic P, Krupovic M, Kuhn JH, Lavigne R, Oksanen HM, Sullivan MB, Jang HB, Simmonds P, Aiewsakun P, Wittmann J, Tolstoy I, Brister JR, Kropinski AM, Adriaenssens EM. 2020. Analysis of spounaviruses as a case study for the overdue reclassification of tailed phages. Syst Biol 69:110–123. doi:10.1093/sysbio/syz036 31127947 PMC7409376

[33] Cao J, Wang C, Zhang Y, Lei G, Xu K, Zhao N, Lu J, Meng F, Yu L, Yan J, Bai C, Zhang S, Zhang N, Gong Y, Bi Y, Shi Y, Chen Z, Dai L, Wang J, Yang P. 2021. Integrated gut virome and bacteriome dynamics in COVID-19 patients. Gut Microbes 13:1–21. doi:10.1080/19490976.2021.1887722 PMC7946006 33678150

[34] Lu ZH, Zhou HW, Wu WK, Fu T, Yan M, He Z, Sun SW, Ji ZH, Shao ZJ. 2021. Alterations in the composition of intestinal DNA virome in patients with COVID-19. Front Cell Infect Microbiol 11:790422. doi:10.3389/fcimb.2021.790422 34900762 PMC8653907

[35] Zuo T, Liu Q, Zhang F, Yeoh YK, Wan Y, Zhan H, Lui GCY, Chen Z, Li AYL, Cheung CP, Chen N, Lv W, Ng RWY, Tso EYK, Fung KSC, Chan V, Ling L, Joynt G, Hui DSC, Chan FKL, Chan PKS, Ng SC. 2021. Temporal landscape of human gut RNA and DNA virome in SARS-CoV-2 infection and severity. Microbiome 9:91. doi:10.1186/s40168-021-01008-x 33853691 PMC8044506

[36] André AC, Debande L, Marteyn BS. 2021. The selective advantage of facultative anaerobes relies on their unique ability to cope with changing oxygen levels during infection. Cell Microbiol 23:e13338. doi:10.1111/cmi.13338 33813807

[37] Lu Z, Imlay JA. 2021. When anaerobes encounter oxygen: mechanisms of oxygen toxicity, tolerance and defence. Nat Rev Microbiol 19:774–785. doi:10.1038/s41579-021-00583-y 34183820 PMC9191689

[38] Hernández S, Vives MJ. 2020. Phages in anaerobic systems. Viruses 12:1091. doi:10.3390/v12101091 32993161 PMC7599459

[39] Łoś M, Węgrzyn G. 2012. Pseudolysogeny. Adv Virus Res 82:339–349. doi:10.1016/B978-0-12-394621-8.00019-4 22420857

[40] Brackman G, Coenye T. 2015. Inhibition of quorum sensing in Staphylococcus spp. Curr Pharm Des 21:2101–2108. doi:10.2174/1381612821666150310101014 25760342

[41] Knowles B, Silveira CB, Bailey BA, Barott K, Cantu VA, Cobián-Güemes AG, Coutinho FH, Dinsdale EA, Felts B, Furby KA, et al.. 2016. Lytic to temperate switching of viral communities. Nature New Biol 531:466–470. doi:10.1038/nature17193 26982729

[42] Silveira CB, Rohwer FL. 2016. Piggyback-the-Winner in host-associated microbial communities. NPJ Biofilms Microbiomes 2:16010. doi:10.1038/npjbiofilms.2016.10 28721247 PMC5515262

[43] Barr JJ, Auro R, Furlan M, Whiteson KL, Erb ML, Pogliano J, Stotland A, Wolkowicz R, Cutting AS, Doran KS, Salamon P, Youle M, Rohwer F. 2013. Bacteriophage adhering to mucus provide a non-host-derived immunity. Proc Natl Acad Sci U S A 110:10771–10776. doi:10.1073/pnas.1305923110 23690590 PMC3696810

[44] Gutiérrez B, Domingo-Calap P. 2020. Phage therapy in gastrointestinal diseases. Microorganisms 8:1420. doi:10.3390/microorganisms8091420 32947790 PMC7565598

[45] Van Belleghem JD, Dąbrowska K, Vaneechoutte M, Barr JJ, Bollyky PL. 2018. Interactions between bacteriophage, bacteria, and the mammalian immune system. Viruses 11:10. doi:10.3390/v11010010 30585199 PMC6356784

[46] Piazzesi A, Pane S, Del Chierico F, Romani L, Campana A, Palma P, Putignani L. 2024. The pediatric gut bacteriome and virome in response to SARS-CoV-2 infection. Front Cell Infect Microbiol 14:1335450. doi:10.3389/fcimb.2024.1335450 38318164 PMC10839054

[47] Ishizaka A, Koga M, Mizutani T, Uraki R, Yamayoshi S, Iwatsuki-Horimoto K, Yamamoto S, Imai M, Tsutsumi T, Suzuki Y, Kawaoka Y, Yotsuyanagi H. 2023. Research article antibody induction and immune response in nasal cavity by third dose of SARS-CoV-2 mRNA vaccination. Virol J 20:146. doi:10.1186/s12985-023-02113-z 37443091 PMC10339591

[48] Bolyen E, Rideout JR, Dillon MR, Bokulich NA, Abnet CC, Al-Ghalith GA, Alexander H, Alm EJ, Arumugam M, Asnicar F, et al.. 2019. Reproducible, interactive, scalable and extensible microbiome data science using QIIME 2. Nat Biotechnol 37:852–857. doi:10.1038/s41587-019-0209-9 31341288 PMC7015180

[49] Callahan BJ, McMurdie PJ, Rosen MJ, Han AW, Johnson AJA, Holmes SP. 2016. DADA2: high-resolution sample inference from Illumina amplicon data. Nat Methods 13:581–583. doi:10.1038/nmeth.3869 27214047 PMC4927377

[50] Quast C, Pruesse E, Yilmaz P, Gerken J, Schweer T, Yarza P, Peplies J, Glöckner FO. 2013. The SILVA ribosomal RNA gene database project: improved data processing and web-based tools. Nucleic Acids Res 41:D590–D596. doi:10.1093/nar/gks1219 23193283 PMC3531112

[51] Fabian Pedregosa GV, Gramfort A, Michel V, Thirion B, Grisel O, Blondel M, Müller A, Nothman J, Louppe G, Prettenhofer P, Weiss R, Dubourg V, Vanderplas J, Passos A, Cournapeau D, Brucher M, Perrot M, Duchesnay É. 2011. Scikit-learn: machine learning in Python. J Mach Learn Res 12:2825–2830. doi:10.48550/arXiv.1201.0490

[52] Bolger AM, Lohse M, Usadel B. 2014. Trimmomatic: a flexible trimmer for Illumina sequence data. Bioinformatics 30:2114–2120. doi:10.1093/bioinformatics/btu170 24695404 PMC4103590

[53] Schmieder R, Edwards R. 2011. Quality control and preprocessing of metagenomic datasets. Bioinformatics 27:863–864. doi:10.1093/bioinformatics/btr026 21278185 PMC3051327

[54] Nurk S, Meleshko D, Korobeynikov A, Pevzner PA. 2017. metaSPAdes: a new versatile metagenomic assembler. Genome Res 27:824–834. doi:10.1101/gr.213959.116 28298430 PMC5411777

[55] Shen W, Le S, Li Y, Hu F. 2016. SeqKit: a cross-platform and ultrafast toolkit for FASTA/Q file manipulation. PLoS One 11:e0163962. doi:10.1371/journal.pone.0163962 27706213 PMC5051824

[56] Camacho C, Coulouris G, Avagyan V, Ma N, Papadopoulos J, Bealer K, Madden TL. 2009. BLAST+: architecture and applications. BMC Bioinformatics 10:421. doi:10.1186/1471-2105-10-421 20003500 PMC2803857

[57] Kieft K, Zhou Z, Anantharaman K. 2020. VIBRANT: automated recovery, annotation and curation of microbial viruses, and evaluation of viral community function from genomic sequences. Microbiome 8:90. doi:10.1186/s40168-020-00867-0 32522236 PMC7288430

[58] Guo J, Bolduc B, Zayed AA, Varsani A, Dominguez-Huerta G, Delmont TO, Pratama AA, Gazitúa MC, Vik D, Sullivan MB, Roux S. 2021. VirSorter2: a multi-classifier, expert-guided approach to detect diverse DNA and RNA viruses. Microbiome 9:37. doi:10.1186/s40168-020-00990-y 33522966 PMC7852108

[59] Ren J, Ahlgren NA, Lu YY, Fuhrman JA, Sun F. 2017. VirFinder: a novel k-mer based tool for identifying viral sequences from assembled metagenomic data. Microbiome 5:69. doi:10.1186/s40168-017-0283-5 28683828 PMC5501583

[60] Clooney AG, Sutton TDS, Shkoporov AN, Holohan RK, Daly KM, O’Regan O, Ryan FJ, Draper LA, Plevy SE, Ross RP, Hill C. 2019. Whole-virome analysis sheds light on viral dark matter in inflammatory bowel disease. Cell Host Microbe 26:764–778. doi:10.1016/j.chom.2019.10.009 31757768

[61] Chao A, Jost L. 2012. Coverage-based rarefaction and extrapolation: standardizing samples by completeness rather than size. Ecology 93:2533–2547. doi:10.1890/11-1952.1 23431585

