# Supplementary material for: Dysbiosis of gut microbiota in COVID-19 is associated with intestinal DNA phage dynamics of lysogenic and lytic infection
Source: Microbiol Spectr. 2024 Dec 10;13(1):e00998-24. doi: 10.1128/spectrum.00998-24 (PMC11705802; doi:10.1128/spectrum.00998-24)
Supplement: Supplemental figures — Fig. S1 and S2. [file spectrum.00998-24-s0001.pdf]

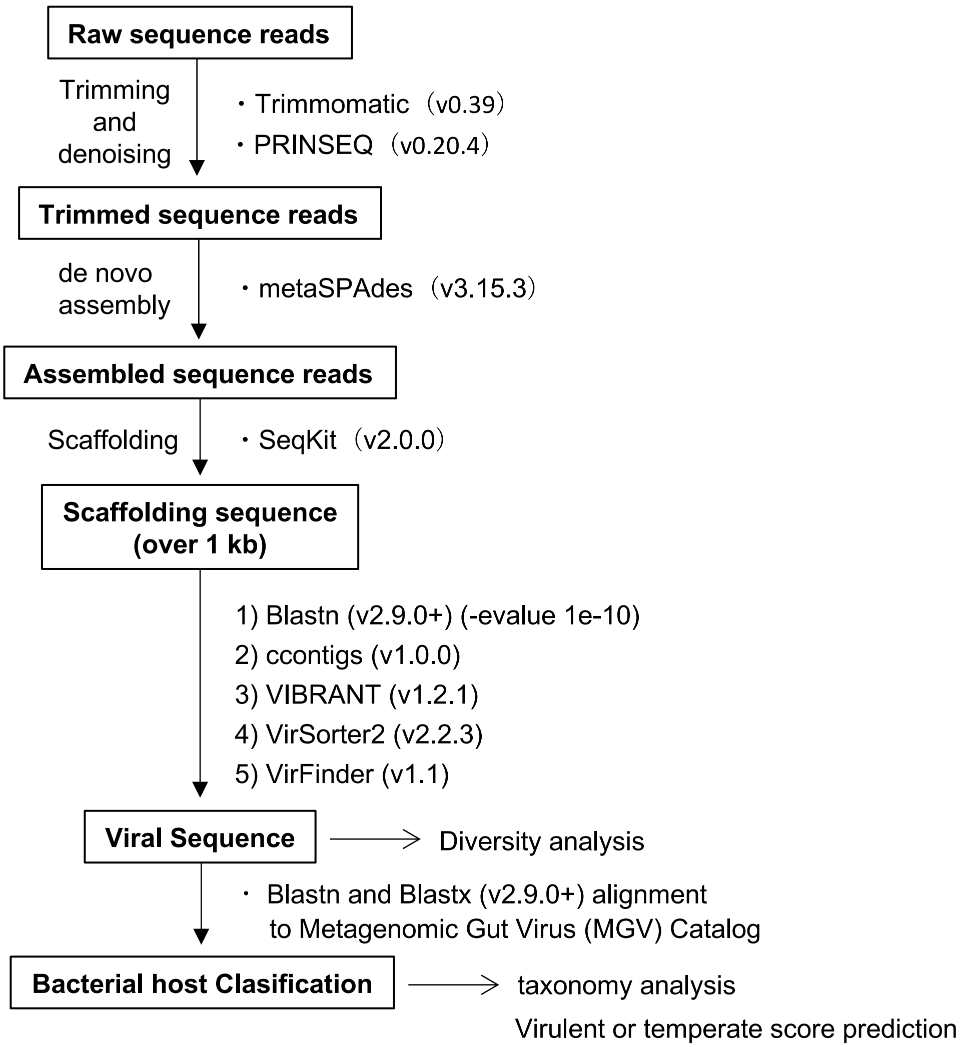

Figure S1 Pipeline and flowchart of intestinal DNA phage analysis

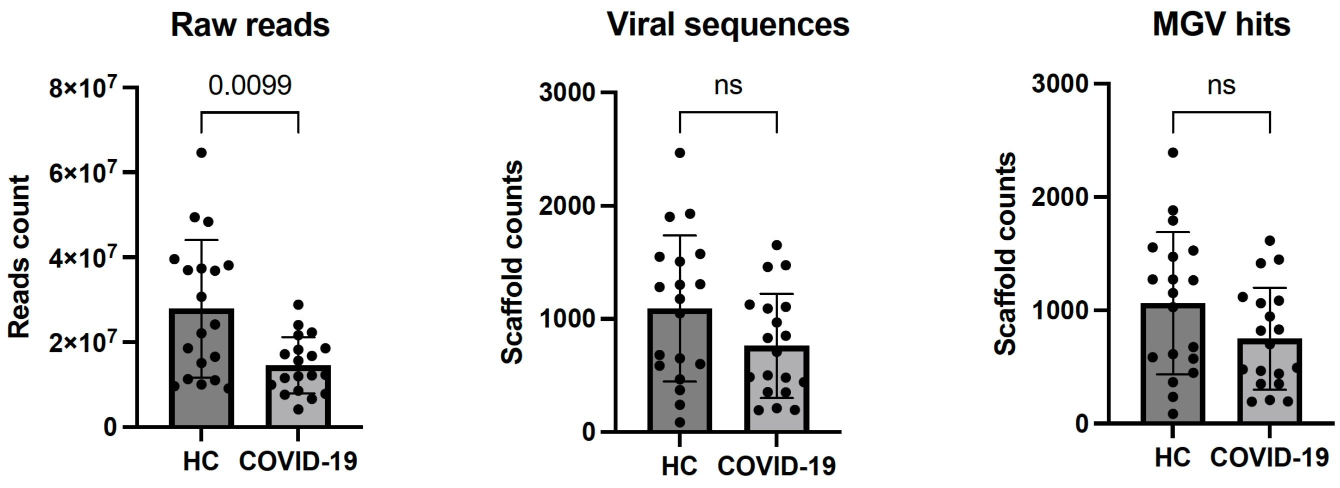

Figure S2 Comparative analysis of raw sequence reads, viral sequence counts and MGV hits between samples (HC and COVID-19 patient groups). Mann-Whitney test was used for two-group comparisons. P value is shown. ns; not significant
